# A Large Voltage Responsivity Pyroelectric Sensor Based on Hot-Pressed Lead Zirconate Titanate Ceramic

**DOI:** 10.3390/s25030917

**Published:** 2025-02-03

**Authors:** Yanhao Guo, Shaobo Guo, Chunhua Yao, Zhiwei Pan, Genshui Wang

**Affiliations:** 1 School of Chemistry and Materials Science, Hangzhou Institute for Advanced Study, University of Chinese Academy of Sciences, Hangzhou 310024, China; guoyanhao22@mails.ucas.ac.cn; 2 Key Laboratory of Inorganic Functional Materials and Devices, Shanghai Institute of Ceramics, Chinese Academy of Sciences, 588 Heshuo Road, Jiading District, Shanghai 201800, China; guoshaobo@mail.sic.ac.cn (S.G.); yaochunhua@mail.sic.ac.cn (C.Y.); panzhiwei@mail.sic.ac.cn (Z.P.)

**Keywords:** PZT ceramics, hot pressed, pyroelectric sensor, current mode, voltage responsivity

## Abstract

In this article, hot-pressed PZT ceramics were used as a sensitive element material and made into a pyroelectric chip. Three current mode sensors were fabricated using a pyroelectric chip of different thicknesses (80 μm, 40 μm, and 30 μm). The voltage responsivity of sensors reached the order of magnitude of 10^5^. The size effect resulting from varying the thickness was studied. The results indicate that as the thickness decreases, the performance significantly increases. When the modulation frequency is 10 Hz, the specific detectivity of the sensor with a 30 μm PZT ceramic pyroelectric chip reaches 5.3 × 10^8^ cm·Hz^1/2^/W.

## 1. Introduction

Proposing using pyroelectricity to fabricate infrared sensors dates back to 1938 [1]. These sensors were originally conceived for use in spectroscopy. The first fast IR pyroelectric sensor using BaTiO_3_ was made by J. Cooper in 1962 [2]. Lithium tantalate (LT) crystals, triglycine sulfide (TGS) crystals, and lead zirconate titanate (PZT) ceramics are the three most commonly used commercial pyroelectric materials. With the rise of the Internet of Things, there will be an increasing demand for electronic devices and sensors. Owing to the advantages of not requiring additional cooling equipment and having low power, low cost, and a fast response [3,4,5,6,7], pyroelectric sensors have been widely used in security monitoring, industrial testing, public safety, energy, smart homes, and medical fields [8,9,10].

Most manufacturers use a single LT single as the pyroelectric sensor material due to its high Curie temperature above 600 °C and small relative dielectric constant [11,12]. However, its pyroelectric coefficient is small, obstructing the magnitude of the current of the sensors. TGS has a large pyroelectric coefficient of 5.5 × 10^−4^ Cm^−2^K^−1^, but its low Curie temperature and water solubility limit its development [13]. The pyroelectric coefficient of PZT ceramics could increase more than twice that of single LT crystals, and the Curie temperature could exceed 200 °C [14]. However, the performance of such sensors is hard to elevate when used in voltage mode because of the large relative dielectric constant of PZT ceramics. However, in current mode, the electric time constant is independent of the relative dielectric constant of the pyroelectric material. So, the advantages of the large pyroelectric coefficient of PZT materials can be brought into play.

The variation law of voltage responsivity has been studied for a long time [15,16]. For voltage mode sensors, the high-frequency voltage responsivity $\left(R_{V}\right)$ is calculated by Equation (1). $C_{E}$ is the capacitance of the element, $C_{A}$ is the capacitance of the JFET, η is the absorption rate on the upper surface of the pyroelectric chips, p is the pyroelectric coefficient, $C_{v}$ is the volume specific heat, $d_{p}$ is the thickness of the pyroelectric chips, and ω is the modulation frequency.

(1) $$R_{V}=\frac{\eta p}{C_{v}d_{p}(C_{E}+C_{A})\omega }$$

If the $C_{A}$ is much larger compared with $C_{E}$, $R_{V}$ is expressed by Equation (2). ε and $\varepsilon_{0}$ are, respectively, the vacuum dielectric constant and the relative dielectric constant. $A_{s}$ is the area of the pyroelectric chip.

(2) $$R_{V}=\frac{\eta p}{C_{v}\varepsilon \varepsilon_{0}A_{s}\omega }$$

Therefore, the voltage responsivity of voltage mode sensors is related to $F_{V}=p/C_{v}\varepsilon \varepsilon_{0}$ [17]. For current mode, the situation is different. At an appropriate frequency, voltage responsivity conforms to Equation (3). $R_{f}$ is the feedback resistor.

(3) $$R_{V}=\frac{\eta pR_{f}}{C_{v}d_{p}}$$

The current mode pyroelectric sensor has a greater voltage responsivity than the voltage mode sensor [18], and the magnitude of the voltage responsivity can be further improved by reducing the thickness and increasing the resistance of the feedback resistor.

In 1972, R. J. MAHLER et al. [19] demonstrated that PZT ceramic materials can be used to prepare high-performance pyroelectric sensors, with a specific detectivity of up to 7 × 10^8^ cm·Hz^1/2^/W at 1 Hz. Its voltage responsivity is only 0.3 mV/W. The high specific detectivity is due to the lack of subsequent amplification circuits which are common in modern commercial sensors, so the noise mainly comes from Johnson noise. The commercial PZT-4 material they used has a pyroelectric coefficient of 2.7 × 10^−4^ Cm^−2^K^−1^, a relative permittivity of 1300, and a volume heat capacity of 3.192 × 10^6^ Jm^−3^K^−1^. To improve the performance of PZT materials and sensors, O.P. Thakur et al. [20] improved the performance of PZT via doping with samarium (Sm), integrated the sensor with an FET amplifier, and evaluated the performance at different chopping frequencies through compensation components. The maximum specific detectivity reached 2 × 10^8^ cm·Hz^1/2^/W, and the voltage responsivity reached 2 × 10^3^ V/W. The thickness of sensitive elements is an important factor affecting device performance. Compared to bulk devices, ferroelectric thin films have lower thermal capacity and are suitable for high-frequency operation, but their smaller pyroelectric coefficient reduces the specific detectivity. Tan Qiu-lin et al. [21] studied the properties of PZT multilayer films prepared by using the sol–gel method and their applications in infrared gas sensors. Sensor structure designs such as a dual-element structure, microbridge structure, and transition layer are used to reduce common mode noise and thermal loss. With the low pyroelectric coefficient of a PZT thin film, the specific detectivity of the sensor is only 0.4 × 10^8^ cm·Hz^1/2^/W and the voltage responsivity is 42 V/W. The above two devices both use voltage mode, so the influence of the thickness of the pyroelectric chip on device performance has not been studied. According to Equation (2), the voltage responsivity is independent of thickness in voltage mode. Yoshiharu Taniguchi et al. [22] showed that by reducing the thickness of the components to 10 μm, the amplitude and rate of temperature rise can be increased, thereby improving the sensitivity and response speed of the sensor. The voltage responsivity is 3.63 × 10^4^ to 3.79 × 10^4^ V/W, which is five times higher than that of a 100 μm thick PZT ceramic current mode sensor.

In this article, a PZT ceramic material using hot-pressed technology is used as a sensitive element material for sensors. Hot-pressed technology, which ensures that ceramic materials become uniform blocks with excellent mechanical properties, is beneficial for subsequent processing such as cutting, lapping, polishing, and dicing [15]. This is conducive to obtaining a thinner pyroelectric chip to improve the performance of pyroelectric sensors.

A current mode pyroelectric sensor based on PZT ceramic is introduced in this work. The selected hot-pressed PZT material has a high pyroelectric coefficient and low dielectric loss, ensuring the performance of the sensor in current mode. Two copper square columns are used to support the pyroelectric chip, ensuring that the center of the pyroelectric chip is in a suspended structure, thereby increasing the pyroelectric current. The reduction in thickness will further increase the pyroelectric current, and three sensors with a pyroelectric chip of different thicknesses (80 μm, 40 μm, and 30 μm) are compared. When the modulation frequency is 10 Hz, a large voltage responsivity of 3.7 × 10^5^ V/W is obtained, and the specific detectivity of the 30 μm-sensor reaches 5.3 × 10^8^ cm·Hz^1/2^/W.

## 2. Materials and Methods

Lead zirconate titanate (PZT) ceramic sheets with an area of 22 × 22 mm^2^ and thicknesses of 80 μm, 40 μm, and 30 μm are selected as the pyroelectric material. The physical properties of the PZT ceramic are listed in Table 1

**Figure 1:**
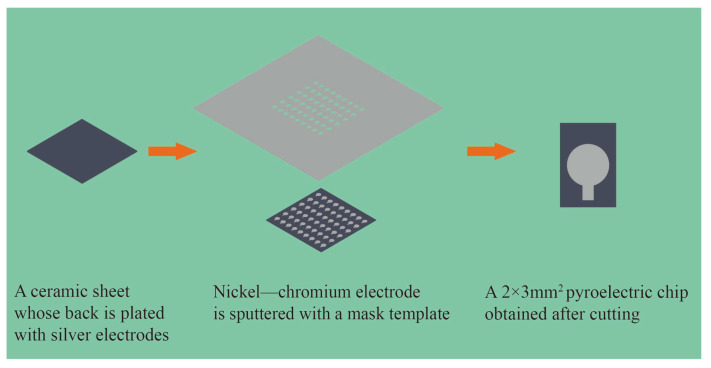
Preparation process of pyroelectric chip.

In advance, silver is sputtered onto one side of the ceramic sheets as the lower electrode. A mask template is designed for sputtering the upper electrode, as shown in Figure 1**.** The nickel–chromium electrode is sputtered as the upper electrode on the other side of the ceramic sheets, and then the ceramic sheets are cut into small pyroelectric chips of 2 × 3 mm^2^. The area of the upper electrode is a circle with a diameter of 1.5 mm, with a wiring position reserved.

The physical printed circuit board is manufactured by Shenzhen JLC Technology Group Co., Ltd. (Zhuhai city, China). Low-noise OPA1671 operational amplifiers (Op Amps) from Texas Instruments (Dallas, TX, USA) are used in the sensors. The value of the capacitance ($C_{f}$) and resistance ($R_{f}$) in the feedback network is 0.2 pF and 75 GΩ, respectively. To support the pyroelectric chip and connect the lower electrode of the pyroelectric chip with the PCB, three 1 × 1 × 1 mm^3^ copper square columns are used.

**Figure 2:**
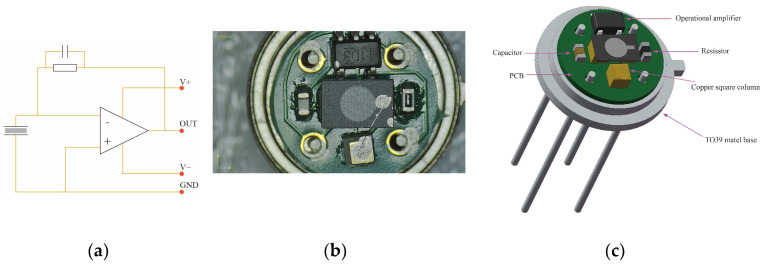
(**a**) Schematic diagram of current mode pyroelectric sensor; (**b**) physical picture of pyroelectric sensor; (**c**) 3D simulation diagram of pyroelectric sensor.

The resistors, capacitors, Op Amps, and 1 × 1 × 1 mm^3^ square columns are soldered onto the PCB using solder paste. The PCB is securely attached to the TO-39 metal base with anaerobic glue, while the pin of the metal base is connected to the PCB using silver paste. The lower electrode of the pyroelectric chip on the PCB is connected to a copper square column with silver paste; the upper electrode of pyroelectric chip is connected by a metal wire to the third copper hexahedron using silver paste. Finally, the device undergoes one hour of heating at a temperature of 150 °C on a hot plate to complete its production. The three different pyroelectric infrared sensors with different thicknesses are named 80 μm-sensor, 40 μm-sensor, and 30 μm-sensor. A physical picture, schematic diagram, and 3D simulation diagram are shown in Figure 2.

**Figure 3:**
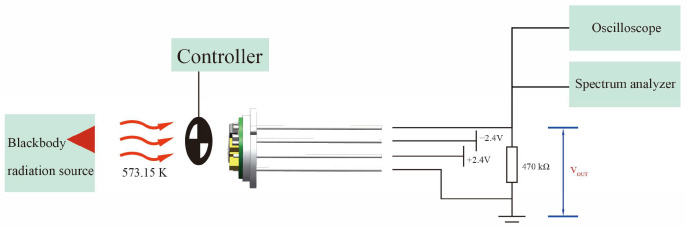
The pyroelectric blackbody sensor test system.

The absorbance of the nickel–chromium electrode and PZT is measured by a Fourier transform IR (FTIR) spectrometer (EQUINOX55, Bruker Co., Germany, Karlsruhe, German). The pyroelectric blackbody sensor test system is set up to test the performance of the prepared sensor, and the test system is shown in Figure 3. The main instruments include a blackbody radiation source (HFY-205A, Shanghai Fuyuan Photoelectric Technology Co., Ltd., Shanghai, China), an optical chopper (OE3001, Guangzhou Sine Scientific Instrument Co., Ltd., Guangzhou, China), an oscilloscope (TBS1102, Tektronix, Inc., Beaverton, Box, OR, USA), a spectrum analyzer (SR770 Systems Research Laboratory, Inc., Sunnyvale, CA, USA), and a self-made test circuit board. The blackbody radiation source emits blackbody radiation at 573.15 K, which is subsequently modulated into 1 Hz to 100 Hz by the optical chopper. The modulated blackbody radiation approximates a square wave, and the power of the modulated blackbody radiation irradiated onto the measured pyroelectric sensor is 1.50 × 10^−6^ W. The pyroelectric signal is transmitted to the test circuit board. Then, the oscilloscope and the spectrum analyzer are used to measure the signal peak–peak value ($V_{pp}$), the effective signal value ($V_{rms}$), and the noise density ($V_{noise}$).

## 3. Results and Discussion

**Figure 4:**
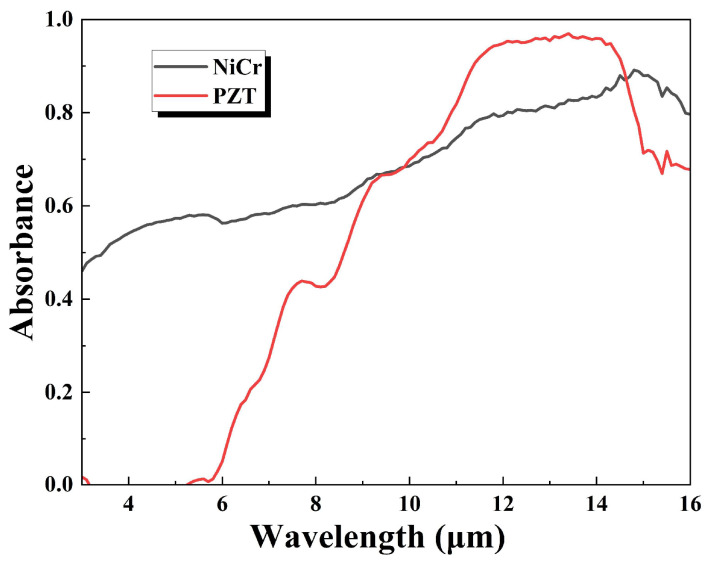
Absorption spectrum of nickel–chromium electrode and PZT.

As shown in Figure 4, the absorbance of the nickel–chromium electrode for wavelengths from 3 μm to 16 μm is mostly above 0.5, with a maximum of 0.89. The PZT material also contributes to infrared absorption, with an absorbance of over 0.9 for infrared light at wavelengths from 11 μm to 15 μm.

**Figure 5:**
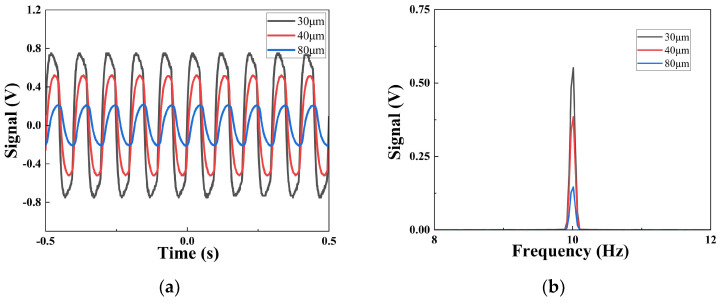
Pyroelectric signal with different thicknesses at 10 Hz: (**a**) peak–peak value of sensors with different thicknesses in time domain at 10 Hz; (**b**) effective signal value of sensors with different thicknesses in frequency domain at 10 Hz.

Figure 5a shows the $V_{pp}$ of the 10 Hz current mode pyroelectric infrared sensors with different thicknesses in the time domain. The size of the value of $V_{pp}$ clearly increases with the decrease in the thickness of the sensitive element. When the thicknesses are 80 μm, 40 μm, and 30 μm, respectively, the values are 0.42 V, 1.04 V, and 1.50 V. The size of the $V_{pp}$ at 10 Hz of the 30 μm-sensor is 3.57 times as big as the size of the 80 μm-sensor. Compared to the time domain, the $V_{rms}$ in the frequency domain is measured using a spectrum analyzer. Figure 5b shows the effective signal value of the sensors with different thickness in the frequency domain at 10 Hz. As expected, the size of the $V_{rms}$ also increases with the decrease in the sensitive element’s thickness. The $V_{rms}$ increases from 0.15 V to 0.55 V.

As the thickness of the pyroelectric chip decreases, the heat capacity (H) of the pyroelectric chip become smaller, resulting in greater temperature fluctuations (∆T). The increase in temperature is related to incident power density according to Equations (4)–(6) [1,15].

(4) $$H=A_{s}C_{v}d_{p}$$

(5) $$G_{T}=g_{T}A_{s}$$

(6) $$∆T=\frac{\eta W(t)}{G_{T}+j\omega H}$$

where H is the heat capacity of the pyroelectric chip, $C_{v}$ is the volumetric specific heat, $G_{T}$ is the heat conductance of the pyroelectric material which is assumed to be proportional to $A_{s}$ with $g_{T}$ [1], η is the absorption rate on the upper surface of the pyroelectric chip, and W(t) is the power density of radiation.

**Figure 6:**
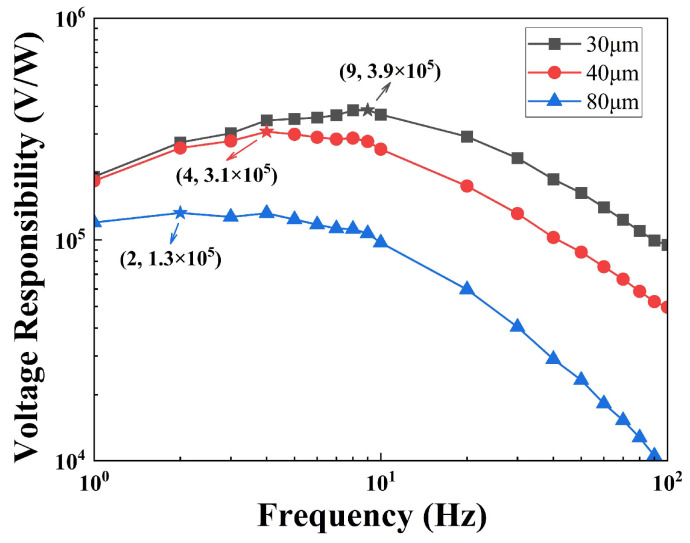
Voltage responsivity dependence on frequency of sensors with different thicknesses.

The detection performance of the sensors within the modulation frequency range of 1 to 100 Hz is investigated. The voltage responsivity ($R_{v}$) is calculated by Equation (7).

(7) $$R_{v}=\frac{V_{rms}}{P}$$

where P is the power of the blackbody radiation on the pyroelectric chip. Figure 6 shows the value of the $R_{v}$ dependence on the frequency of the pyroelectric infrared sensors. As the thickness decreases, the voltage responsivity increases, and the frequency of the corresponding maximum voltage responsivity increases. For the 80 μm-sensor, the maximum voltage responsivity is 1.3 × 10^5^ V/W at 2 Hz; for the 40 μm-sensor, the maximum voltage responsivity is 3.1 × 10^5^ V/W at 4 Hz; and for the 30 μm-sensor, the maximum voltage responsivity is 3.9 × 10^5^ V/W at 9 Hz.

As the thickness decreases, H decreases. The thermal time constant ($\tau_{T}$) also decreases, and the thermal corner frequency ($f_{T}$) increases. The variation law of $\tau_{T}$ and $f_{T}$ with thickness is expressed by Equations (8) and (9). Therefore, the frequency of the maximum voltage responsivity increases.

(8) $$\tau_{T}=\frac{H}{G_{T}}=\frac{A_{s}C_{v}d_{p}}{G_{T}}\;$$

(9) $$f_{T}=\frac{1}{2\pi \tau_{T}}=\frac{G_{T}}{2\pi A_{s}C_{v}d_{p}}$$

**Figure 7:**
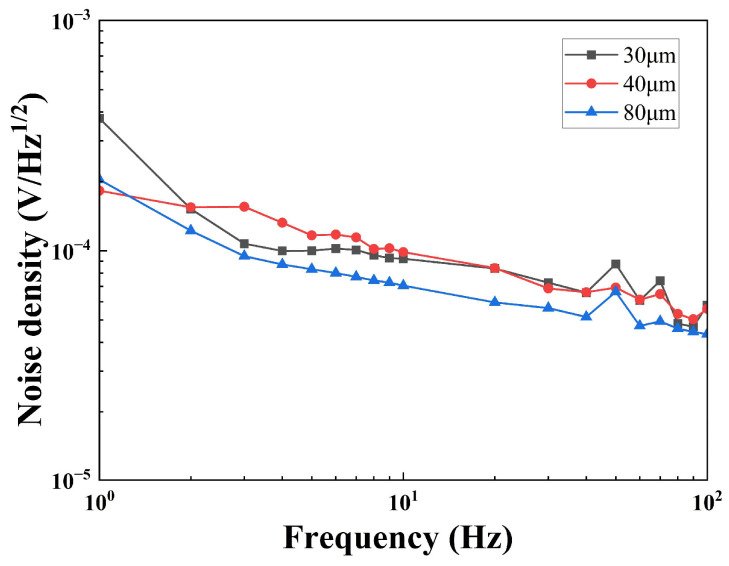
The noise density dependence on the frequency of sensors with different thicknesses.

**Figure 8:**
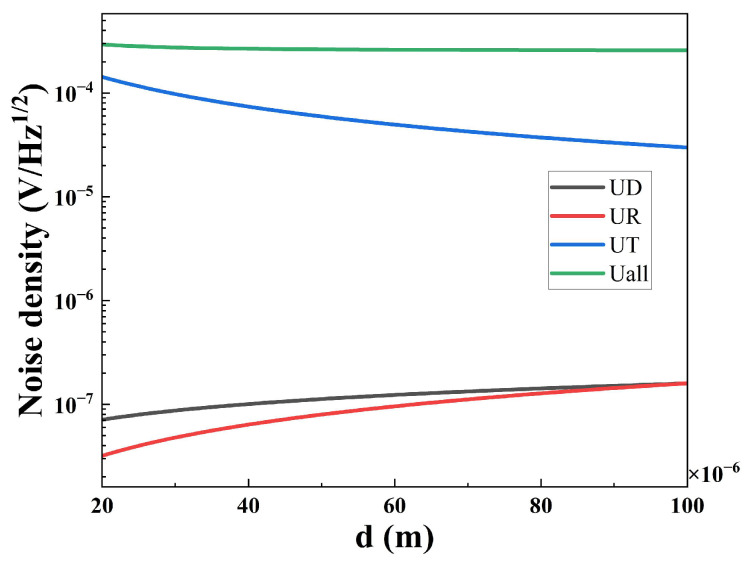
The noise density dependence on different thicknesses (20–100 μm) of a pyroelectric chip at 10 Hz calculated by GNU Octave.

Figure 7 shows the noise density of the sensors with different thicknesses; the 80 μm-sensor has the lowest noise density. The noise density of a current mode pyroelectric sensor includes five types of noise [23]; they are resistance thermal noise ($U_{R}$), dielectric loss noise ($U_{D}$), temperature noise ($U_{T}$), voltage noise ($U_{V}$), and current noise ($U_{I}$). The total noise density ($U_{N}$) is described by Equation (13). Among the five noises, $U_{R}$, $U_{D}$, and $U_{T}$ are dependent on thickness, as Equations (10)–(12) show. Figure 8 shows the noise density dependence on different thicknesses (20–100 μm) of the pyroelectric chip at 10 Hz calculated by GNU Octave [24]. $U_{N}$ becomes bigger as the thickness of the pyroelectric chip becomes smaller, because the increase in $U_{T}$ exceeds the sum of the decrease in $U_{D}$ and $U_{R}$. Therefore, a thinner pyroelectric chip means larger noise.

(10) $$U_{R}={\left(\frac{4k_{B}T}{R_{f}}\right)}^{\frac{1}{2}}\frac{R_{f}}{{\left(1+\omega^{2}\tau_{E}^{2}\right)}^{\frac{1}{2}}}={\left(\frac{4k_{B}T}{R_{f}}\right)}^{\frac{1}{2}}\frac{R_{f}}{{\left(1+\omega^{2}R_{f}^{2}C_{f}^{2}\right)}^{\frac{1}{2}}}$$

(11) $$U_{D}={\left(4k_{B}T\omega C_{p}tan\delta \right)}^{\frac{1}{2}}\frac{R_{f}}{{\left(1+\omega^{2}\tau_{E}^{2}\right)}^{\frac{1}{2}}}={\left(4k_{B}T\omega \frac{\varepsilon_{0}\varepsilon_{r}A_{s}}{d_{p}}tan\delta \right)}^{\frac{1}{2}}\frac{R_{f}}{{\left(1+\omega^{2}R_{f}^{2}C_{f}^{2}\right)}^{\frac{1}{2}}}$$

(12) $$U_{T}={\frac{R_{V}}{\eta }\left(4k_{B}T^{2}G_{T}\right)}^{\frac{1}{2}}=\frac{pA_{s}\omega R_{f}}{G_{T}{\left(1+\omega^{2}{\left(\frac{A_{s}C_{v}d_{p}}{G_{T}}\right)}^{2}\right)}^{\frac{1}{2}}{\left(1+\omega^{2}R_{f}^{2}C_{f}^{2}\right)}^{\frac{1}{2}}}$$

(13) $$U_{N}=\sqrt{{U_{R}}^{2}+{U_{D}}^{2}+{U_{T}}^{2}+{U_{V}}^{2}+{U_{I}}^{2}}$$

**Figure 9:**
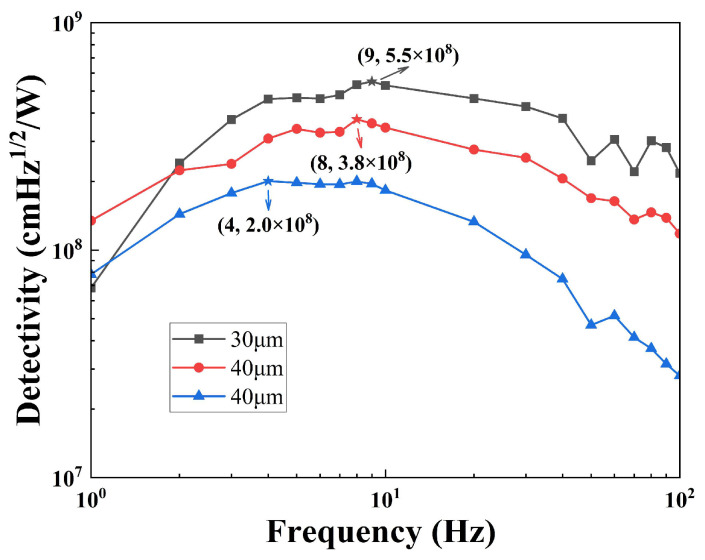
The specific detectivity dependence on the frequency of sensors with different thicknesses.

The specific detectivity is an important parameter that characterizes the performance of sensors, which is calculated by Equation (14). The change law of the specific detectivity is consistent with the voltage responsivity, as shown in Figure 9. For the 30 μm-sensor, the specific detectivity reaches 5.5 × 10^8^ cmHz^1/2^/W at 9 Hz. The 30 μm-sensor is compared with similar commercial sensors, as shown in Table 2; the performance of the sensor is higher than that of the compared sensors.

(14) $$D^{*}=\frac{\sqrt{A_{S}}R_{V}}{U_{N}}$$

## 4. Conclusions

Three current mode sensors were fabricated using a pyroelectric chip of different thicknesses. The electrical time constant is independent of the relative dielectric constant of the sensitive element material and the large gain of the Op Amps in current mode pyroelectric sensors. A large voltage responsivity of the sensors is easily obtained. The size of the voltage responsivity reaches the order of magnitude of 10^5^. A large signal response means that there is no need for amplification circuits in the future, and it can be directly used in conjunction with an analog-to-digital converter. And the price of ceramic materials is better than that of lithium tantalate single crystals, which also provides consumers with a low-cost and high-response choice for pyroelectric sensors. Reducing thickness is beneficial for improving sensor performance. Compared to commercial sensors, the 30 μm-sensor shows good performance among the three fabricated pyroelectric sensors, with its specific detectivity reaching 5.5 × 10^8^ cmHz^1/2^/W at 9 Hz. On the other hand, the absorption rate of infrared light by pyroelectric chips has not reached the optimal effect. In the future, we will improve the infrared absorption layer to further enhance the performance of the sensor.

## Figures and Tables

**Table 1:** The physical properties of the PZT ceramic.

Physical Properties	Value
Pyroelectric coefficient (p)	4.5 × 10^−4^ C/m^2^K
Curie temperature ($T_{c}$)	234 °C
Relative dielectric constant ($\varepsilon_{r}$)	300 (@1 kHz)
Resistivity (ρ)	1.1 × 10^12^ Ω·cm
Dielectric loss (tanδ)	0.0045 (@1 kHz)
Volumetric heat capacity ($C_{v}$)	2.638 × 10^6^ J/m^3^·K

**Table 2:** Comparison of device performance among various companies.

Corporate	Model	Type	Pyroelectric Material	Voltage Responsivity	Specific Detectivity
InfraTec [25]	LME-551	Current mode	LT	6000	2.5 × 10^8^
BROADCOM [26]	AFBR	Current mode	PZT thin films	150,000	3.5 × 10^8^
Excelitas [27]	PYD	Voltage mode	PZT ceramics	42,000	/
This work	30 μm-sensor	Current mode	PZT ceramics	367,700	5.3 × 10^8^

## Data Availability

The data are contained within the article.
